# Allosteric AKT Inhibitors Target Synthetic Lethal Vulnerabilities in E-Cadherin-Deficient Cells

**DOI:** 10.3390/cancers11091359

**Published:** 2019-09-13

**Authors:** Nicola Bougen-Zhukov, Yasmin Nouri, Tanis Godwin, Megan Taylor, Christopher Hakkaart, Andrew Single, Tom Brew, Elizabeth Permina, Augustine Chen, Michael A. Black, Parry Guilford

**Affiliations:** Cancer Genetics Laboratory, Centre for Translational Cancer Research (Te Aho Matatū), Department of Biochemistry, University of Otago, Dunedin 9016, New Zealand; nicola.bougen-zhukov@otago.ac.nz (N.B.-Z.); andrew.single@med.lu.se (A.S.);

**Keywords:** E-cadherin, AKT, diffuse gastric cancer, synthetic lethality, chemoprevention

## Abstract

The *CDH1* gene, encoding the cell adhesion protein E-cadherin, is one of the most frequently mutated genes in gastric cancer and inactivating germline *CDH1* mutations are responsible for hereditary diffuse gastric cancer syndrome (HDGC). Using cell viability assays, we identified that breast (MCF10A) and gastric (NCI-N87) cells lacking *CDH1* expression are more sensitive to allosteric AKT inhibitors than their *CDH1*-expressing isogenic counterparts. Apoptosis priming and total apoptosis assays in the isogenic MCF10A cells confirmed the enhanced sensitivity of E-cadherin-null cells to the AKT inhibitors. In addition, two of these inhibitors, ARQ-092 and MK2206, preferentially targeted mouse-derived gastric *Cdh1*^−/−^ organoids for growth arrest. AKT protein expression and activation (as measured by phosphorylation of serine 473) were differentially regulated in E-cadherin-null MCF10A and NCI-N87 cells, with downregulation in the normal breast cells, but upregulation in the gastric cancer cells. Bioinformatic analysis of the TCGA STAD dataset revealed that *AKT3*, but not *AKT1* or *AKT2*, is upregulated in the majority of E-cadherin-deficient gastric cancers. In conclusion, allosteric AKT inhibitors represent a promising class of drugs for chemoprevention and chemotherapy of cancers with E-cadherin loss.

## 1. Introduction

*CDH1*, encoding the cell–cell adhesion protein E-cadherin [1], is a tumour suppressor gene that is frequently mutated in sporadic diffuse-type gastric cancer (DGC), lobular breast cancer in situ (LCIS), and invasive lobular breast cancer (LBC) [2]. In addition, inactivating germline mutations in *CDH1* are causative of the highly penetrant, inherited cancer syndrome hereditary diffuse gastric cancer (HDGC) [3]. HDGC is characterised by multifocal stage T1a gastric signet ring cell carcinomas in over 95% of mutation carriers from a young age and a 70% lifetime risk of advanced DGC [4]. Female *CDH1* mutation carriers also have a 40% risk of developing LBC [5]. Currently, prophylactic gastrectomy is the recommended option for gastric cancer risk reduction in mutation carriers, despite the high associated morbidity [6].

The frequency of *CDH1* loss in sporadic and familial DGC, LCIS, and LBC suggests that targeting E-cadherin-deficiency in these cancers may provide an effective method for the chemoprevention of HDGC and new treatments for the sporadic disease. This is of particular importance for DGC, which shows a poorer response to many of the currently used chemotherapeutics than the more common intestinal form of gastric cancer (IGC) [7]. Because *CDH1* is a tumour suppressor gene, we have been taking a synthetic lethal approach to identify druggable vulnerabilities created by loss of E-cadherin [8,9]. Synthetic lethality is classically defined as a genetic interaction in which a combination of mutations in two or more genes leads to cell death [10]. In a therapeutic setting, the term can refer to the use of targeted drugs to cause cell death preferentially in tumours carrying specific genetic alterations. By carrying out RNAi and known drug screens on isogenic breast and gastric cell lines with and without E-cadherin, we have identified multiple druggable vulnerabilities in E-cadherin-deficient cells which may be exploited for both the chemoprevention of HDGC and the treatment of sporadic DGC, LCIS, and LBC [8,9]. The screen data has also led to a model in which the principal vulnerability in E-cadherin-deficient cells is disruption of plasma membrane organisation and the associated cytoskeleton, leading to abnormal cell survival signalling, particularly through PI3K/AKT [9]. This model is supported by demonstration that E-cadherin-mediated cell contacts lead to direct signalling through the PI3K/AKT pathway [11], as well as indirect activation of AKT signalling after ligand independent activation of growth factor receptors such as EGFR [12]. Furthermore, proteomic analysis has shown that the AKT and EGFR pathways are upregulated in *CDH1*-mutated gastric tumours [13].

Here, we have searched for inhibitors of PI3K/AKT signalling that show the greatest differential activity against E-cadherin-deficient cells, and tested their synthetic lethal (SL) effect using apoptosis assays and isogenic mouse-derived organoids. Our data show, for the first time, that allosteric AKT inhibitors preferentially promote apoptosis in *CDH1*-deficient cells. We postulate that this susceptibility to allosteric AKT inhibition could be a novel chemopreventative and chemotherapeutic strategy for *CDH1* germline mutation carriers and E-cadherin-negative sporadic cancers, respectively.

## 2. Results

### 2.1. Allosteric AKT Inhibitors Preferentially Target Breast Cells Lacking CDH1

**Figure 1:**
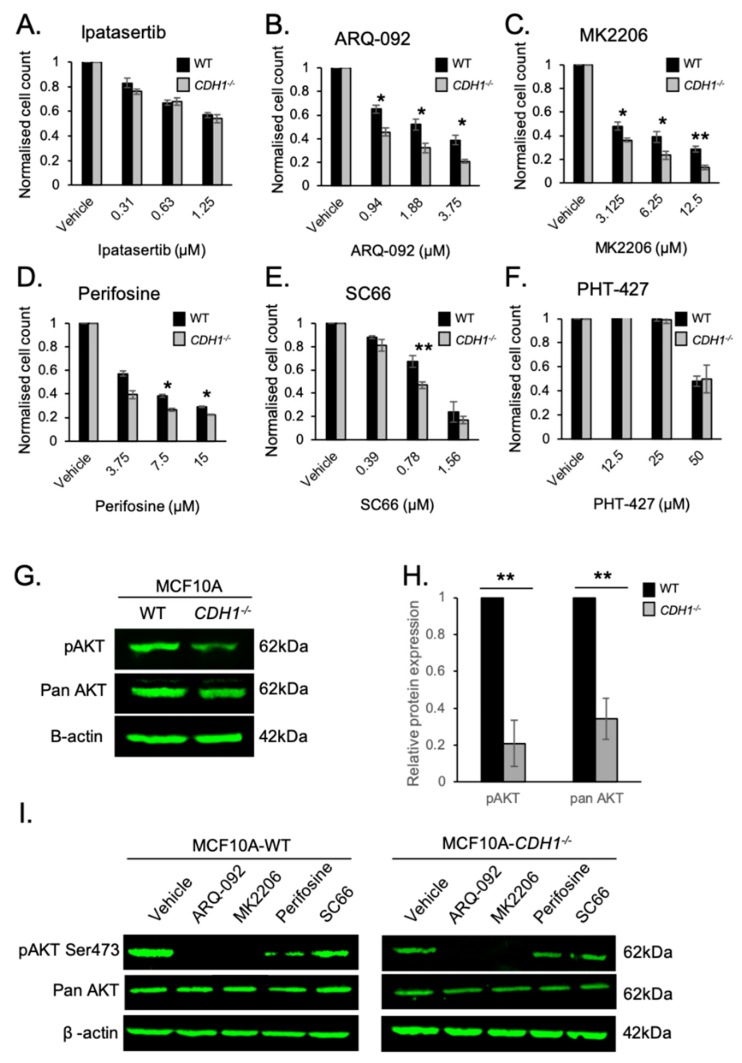
Non-malignant breast cells lacking E-cadherin exhibit a higher sensitivity to the allosteric AKT inhibitors ARQ-092, MK2206, perifosine, and SC66. (**A**–**F**) Normalised MCF10A-WT and *CDH1*^−/−^ cell counts 48 h after treatment with serial dilutions of Ipatasertib (**A**), ARQ-092 (**B**), MK2206 (**C**), Perifosine (**D**), SC66 (**E**), and PHT-427 (**F**). Wild-type, black bars; *CDH1*^−/−^, grey bars. Six fields per well at 4× magnification were captured using the Cytation 5 imager (Biotek). Nuclei were counted using Gen5 (Biotek) and normalised to the vehicle control for each cell line. (**G**) Western blots of pAKT-Ser473 and pan-AKT levels in untreated MCF10A-WT and *CDH1*^−/−^ cells. (**H**) Relative expression of pAKT and pan-AKT in MCF10A-WT and *CDH1*^−/−^ cells. (**I**) Western blots of pAKT-Ser473 and pan-AKT levels in MCF10A-WT and *CDH1*^−/−^ cells after 4 h of DMSO or allosteric AKT inhibitor treatments; vehicle (0.1% DMSO), ARQ092 (2 µM), MK2206 (6.25 µM), perifosine (7.5 µM), and SC66 (0.78 µM). (For all graphs, error bars = SEM; ns = *p* > 0.05, * = *p* < 0.05, ** = *p* < 0.01, *n* ≥ 3 independent biological replicates; unpaired two-sided *t*-test.)

A previous drug screen conducted in our laboratory identified the ATP-competitive AKT inhibitor AZD5363 as a drug which could preferentially slow the growth of MCF10A (non-malignant breast) and NCI-N87 (gastric cancer) cells that are deficient in E-cadherin [9]. For this assay, we utilised a previously characterised pair of isogenic cell lines: MCF10A-WT (containing wild-type (WT) *CDH1*) and MCF10A-*CDH1*^−/−^ (containing a deletion in the *CDH1* locus) (Supplementary Figure S1A) [14]. This cell line was employed to model the effect of *CDH1* deletion on normal (breast) cell response to chemopreventive strategies. Initially, we tested another ATP-competitive AKT inhibitor, Ipatasertib [15]; however, no difference was seen between WT and *CDH1*^−/−^ cell survival after this treatment (Figure 1A). Given the abundance of AKT inhibitors in development for cancer treatment [16], we expanded our screen to include allosteric AKT inhibitors, a newer class of inhibitors that are postulated to have enhanced specificity, reduced side effects, and lower toxicity than other classes of AKT inhibitors [17]. The allosteric AKT inhibitors used in this study, ARQ-092 [18], MK2206 [19], perifosine [20], SC66 [21], and PHT-427 [22], can bind directly to AKT to inhibit its activation and downstream signalling. Importantly, allosteric AKT inhibitors (unlike the ATP-competitive class) do not cause hyperphosphorylation of AKT as a treatment-dependent compensation of signalling [23].

After 48 h treatment with a range of (drug-dependent) concentrations, the MCF10A-*CDH1*^−/−^ cells exhibited a greater sensitivity to the four allosteric AKT inhibitors ARQ-092, MK2206, perifosine, and SC66 (Figure 1B–E), confirming a vulnerability of *CDH1*-deficient cells to inhibition of AKT. No significant difference was seen in cells treated with PHT-427, a dual AKT/PDK1 allosteric inhibitor [24] (Figure 1F).

We next examined both the basal and post-drug treatment protein levels of phosphorylated AKT (phospho-Ser473) and total AKT in the isogenic MCF10A cell lines. Interestingly, basal levels of both p-AKT (Ser473) and pan-AKT were significantly lower in MCF10A-*CDH1*^−/−^ cells compared with WT cells (Figure 1G). It was more difficult to detect phospho-473 in MCF10A-*CDH*^−/−^ cells as they expressed ~20% less of the amount in MCF10A-WT cells (Figure 1H). Pan-AKT protein levels in *CDH1*^−/−^ cells were >60% less than WT (Figure 1H). pAKT and pan-AKT protein levels were also measured in the isogenic cell lines after treatments with the four allosteric inhibitors identified as synthetic lethal candidates in Figure 1B–E. After 4 h treatment with ARQ-092 (2 µM) and MK2206 (6.25 µM), phosphorylation of AKT on serine 473 was completely inhibited in both cell lines. Treatment with perifosine (7.5 µM) slightly reduced pAKT in both cell lines. SC66 (0.78 µM) treatment appeared to have a limited effect on pAKT in either cell line after 4 h treatment. SC66 has been demonstrated to enhance ubiquitinylation and subsequent proteosomal degradation of AKT, thus providing an alternative method for growth inhibition effects seen in these cells [21]. None of the drugs tested had a detectable effect on pan-AKT expression in either cell line.

### 2.2. CDH1-Negative Breast Cells Have Enhanced Apoptotic Priming and Apoptosis Induction after Allosteric AKT Inhibitor Treatment

The results of our nuclei enumeration assay (Figure 1) indicate that non-malignant breast *CDH1*^−/−^ cells are more sensitive to the effects of AKT inhibition. However, this assay simply measures endpoint nuclei number and does not clarify if the reduced number of *CDH1*^−/−^ cells is due to a cytostatic or cytotoxic effect of these drugs. The allosteric AKT inhibitors utilised in this study, such as MK2206, have been shown to induce both apoptosis [25] and/or autophagy [26] in different cell lines.

**Figure 2:**
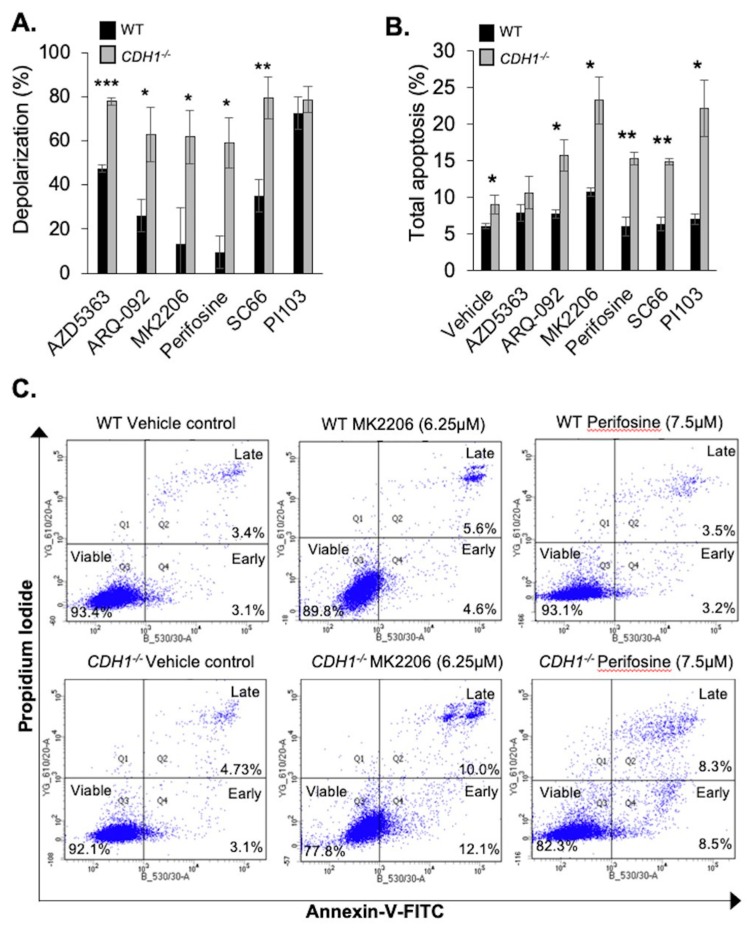
Inhibition of AKT preferentially primes *CDH1*-null cells for apoptosis. (**A**) Mitochondrial membrane depolarisation in MCF10A-WT and *CDH1*^−/−^ cells after 16–20 h treatment with AKT inhibitors. (**B**) Total apoptosis (Annexin-V-FITC and propidium iodide positive cells) detected by flow cytometry after 72 h drug treatment. (**C**) Representative histograms of MCF10A-WT and *CDH1*^−/−^ cells stained with Annexin-V-FITC and propidium iodide and analysed on BD Fortessa flow cytometer. (For all graphs, error bars = SEM; ns = *p* > 0.05, * = *p* < 0.05, ** = *p* < 0.01, *** = *p* < 0.001; *n* ≥ 3 independent biological replicates; unpaired two-sided *t*-test.)

To clarify the potential mechanism by which these small molecule inhibitors preferentially affect *CDH1*^−/−^ cells, we measured the degree of readiness for apoptosis, hereafter referred to as “apoptotic priming”, in our cell lines after 16–20 h of drug treatment. Apoptotic priming was measured by an assay described in Ryan et al. [27], whereby DMSO or drug-treated cells are stained with JC-1 dye and exposed to pro-apoptotic BH3 peptides, such as BIM. If cells are primed for apoptosis, then the exposure to BH3 peptides will lead to mitochondrial outer membrane depolarisation (MOMP) and the JC-1 dye will shift from red aggregates within the mitochondria to green monomers. This decrease in red fluorescence can be used to determine the extent of depolarisation [28]. We observed that all of our AKT inhibitors primed both MCF10A-WT and *CDH1*^−/−^ cells for apoptosis. However, the extent of depolarisation was significantly higher in the *CDH1*^−/−^ cells for all of the inhibitors tested except for the PI3K inhibitor PI103 (Figure 2A). Under the conditions tested, all of the AKT inhibitors (AZD5363, ARQ-092, MK2206, perifosine, and SC66) preferentially primed *CDH1*^−/−^ cells with 20–40% more mitochondrial membrane depolarisation than WT cells (Figure 2A and Supplementary Figure S2A).

To test if the apoptotic priming translated into differences in the induction of apoptosis, we measured apoptosis by flow cytometry analysis of Annexin-V-FITC/Propidium iodide stained cells (Figure 2B) after 72 h treatment. The results of this assay confirmed that the detection of apoptotic priming was generally a good indicator of future apoptosis, with consistent priming and FACS results for all of the allosteric AKT inhibitors tested (Figure 2B). However, despite the ATP-competitive AKT inhibitor AZD5363 (10 µM) leading to almost 80% depolarisation in our *CDH1*^−/−^ cells (~30% more than WT) (Figure 2A and Supplementary Figure S2A), the levels of apoptosis detected in cells treated with this drug for 72 h were not significantly higher than DMSO-treated controls (Figure 2B and Supplementary Figure S2B). However, the allosteric AKT inhibitors all exhibited significantly higher apoptosis induction in MCF10A-*CDH1*^−/−^ cells compared with WT cells (Figure 2E and Supplementary Figure S2C). Out of the AKT inhibitors, MK2206 (6.25 µM) treatment induced the highest level of apoptosis in the MCF10A-*CDH1*^−/−^ cells (20.2%). Interestingly, despite there being no significant difference in apoptotic priming detected between WT and *CDH1*^−/−^ cells after 20 h of PI103 (1 µM) treatment (Figure 2A), at 72 h there was a significantly different level of apoptosis induced: WT 7% vs. *CDH1*^−/−^ 22.2% **, a 3.17-fold difference (Figure 2B and Supplementary Figure S2B,C). Potentially, despite the initiation of priming in MCF10A-WT cells after PI103 treatment, these cells may be more adept than MCF10A-*CDH1*^−/−^ cells in resisting the induction of apoptosis.

### 2.3. CDH1-Negative Gastric Cells Are More Sensitive to the Allosteric AKT Inhibitors ARQ-092 and MK2206

**Figure 3:**
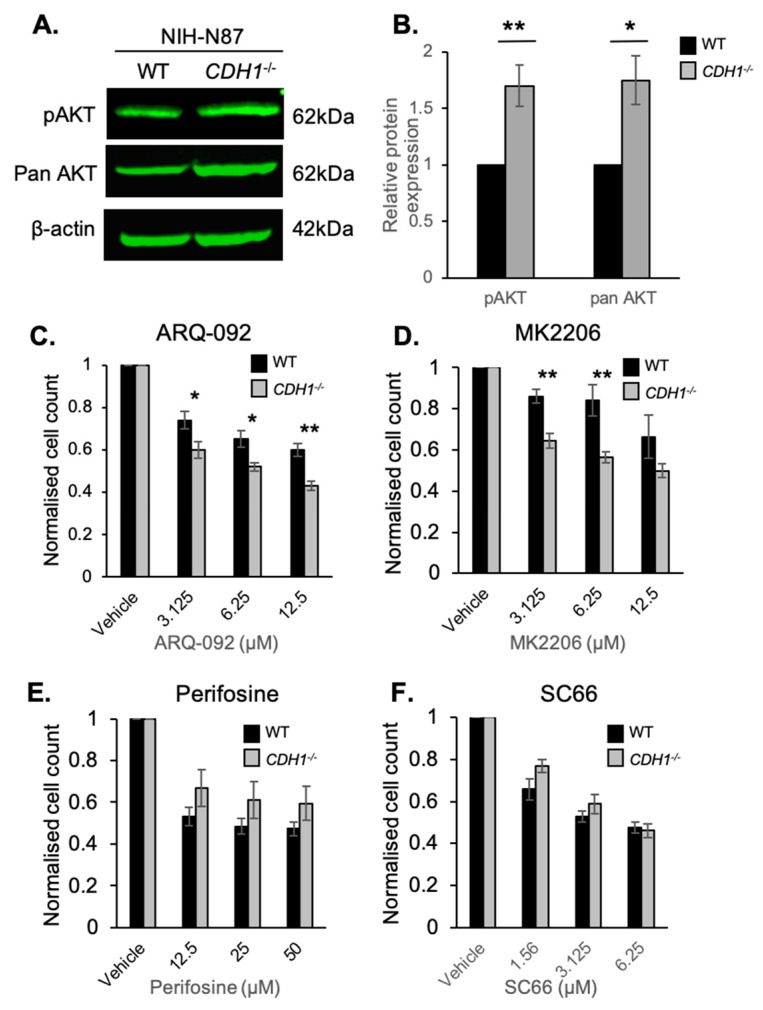
Gastric cancer cells lacking E-cadherin have enhanced sensitivity to the allosteric AKT inhibitors ARQ092 and MK2206. (**A**) Western blots of pAKT-Ser473, pan-AKT, and β-actin in NCI-N87-WT and *CDH1*^−/−^ cells. (**B**) Relative expression of pAKT and pan-AKT in NCI-N87-WT and *CDH1*^−/−^ cells. (**C**–**F**) Normalised NCI-N87-WT and *CDH1*^−/−^ cell counts 48 h after treatment with serial dilutions of ARQ-092 (**C**), MK2206 (**D**), perifosine (**E**), and SC66 (**F**). Wild-type, black bars; *CDH1*^−/−^, grey bars. Six fields per well at 4× magnification were captured using the Cytation 5 imager (Biotek). Nuclei were counted using Gen5 (Biotek, Winooski, Vermont) and normalised to the vehicle control for each cell line. (For all graphs, error bars = SEM; ns = *p* > 0.05, * = *p* < 0.05, ** = *p* < 0.01, *n* ≥ 3 independent biological replicates; unpaired two-sided *t*-test.)

To determine if the synthetic lethal (SL) relationship identified between *CDH1* and allosteric inhibition of AKT was present in cancer cell lines, we tested the allosteric AKT inhibitors in an isogenic cell line pair derived from NCI-N87 gastric cancer cells: NCI-N87-WT and NCI-N87-*CDH*^−/−^ (Supplementary Figure S1B). Prior to drug testing, we measured the basal expression of pAKT (Serine 473) and total AKT (Figure 3A). Surprisingly, unlike the MCF10A isogenic cell lines, the NCI-N87 cells lacking *CDH1* had 1.7-fold higher basal levels of pAKT than NCI-N87-WT cells (Figure 3B). Pan-AKT levels were also increased (1.75-fold) in the NCI-N87-*CDH1*^−/−^ cells compared with WT (Figure 3B). These cells were generally more resistant than the MCF10A cells to the AKT inhibitors, with increased IC_50_ values for all of the drugs tested (Figure 3C–F). For example, the ARQ-092 IC_50_ in MCF10A-WT cells was ~1.88 µM (Figure 1B) vs. ~25 µM in NCI-N87-WT cells (Figure 3C). Importantly, we observed a strong SL effect after treatment with ARQ-092 and MK2206 in the NCI-N87-*CDH1*^−/−^ cell line (Figure 3C,D); however, no such effect was seen after perifosine or SC66 treatment (Figure 3E,F). Since the SL effect of ARQ-092 and MK2206 was consistent across two cell lines from different tissues (mammary vs. gastric) and of different disease states (normal vs. cancer), we decided to take these drugs forward into our gastric organoid model.

### 2.4. Allosteric AKT Inhibitors Preferentially Slow the Growth of Cdh1-Deleted Organoids

To address the inherent limitations of two-dimensional (2D) cell culture models, such as cell population homogeneity and lack of three-dimensional (3D) structures, we next tested our top candidate drugs (ARQ-092 and MK2206) in mouse-derived gastric organoids. Organoids are 3D structures comprised of both differentiated and stem cells, which in part recapitulate the organisation and function of target organs. Propagated in vitro, these spherical cell cultures have the delicate organisation of the in vivo gastric gland, albeit on a simpler scale. Our organoids contained a *CD44*-Cre/*Cdh1*^(fl/fl)^/TdTomato construct, which allows for the inducible cellular knockout of *Cdh1* upon treatment with endoxifen, resulting in organoids containing a mixture of *Cdh1*-null and positive cells, hereafter referred to as *Cdh1*-deleted organoids. This mixture has similarities to the stomachs of germline *CDH1* mutation carriers, whereby discrete regions of E-cadherin-negative gastric cells result in signet ring cell carcinoma formation in a greater milieu of E-cadherin-positive cells. WT organoids displayed uniform expression of E-cadherin (Supplementary Figure S3). We confirmed the percentage of organoid cells with deleted *Cdh1* by staining organoids with an antibody specific for E-cadherin. After induction, organoids had an average of 73% *Cdh1* homozygous deletion per organoid (Supplementary Figure S4).

**Figure 4:**
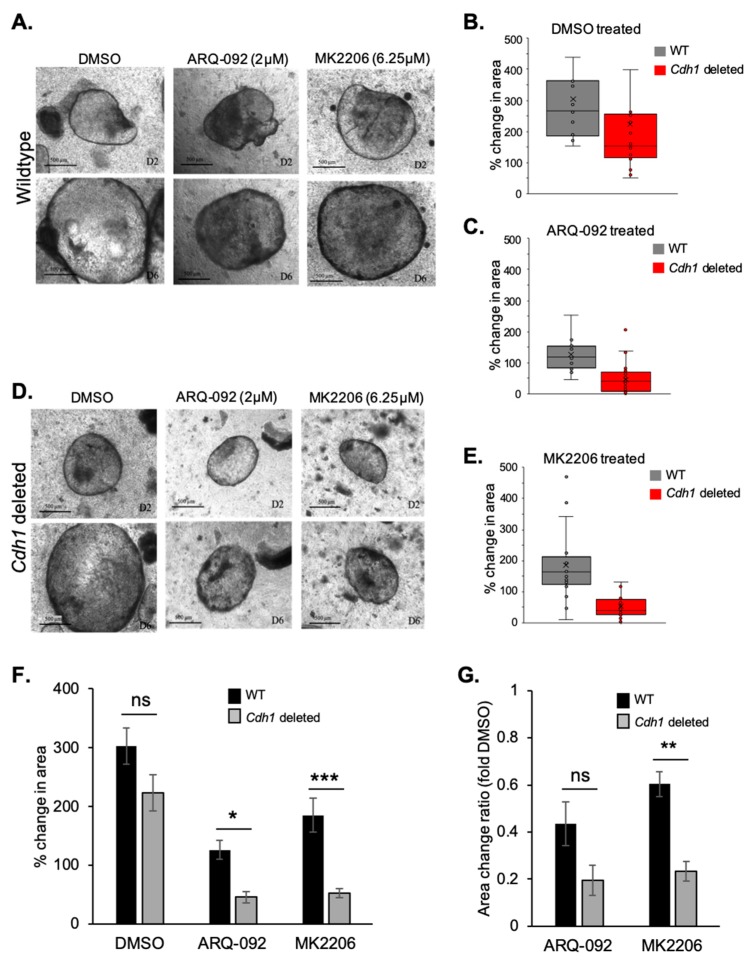
Mouse-derived gastric organoids containing *Cdh1*-null cells are more sensitive to the growth-inhibiting effects of allosteric AKT inhibitors. (**A**) Representative photos of wild-type (WT) organoids exposed to DMSO, ARQ-092, or MK2206 on day 2 (top panel) and day 6 (lower panel) of growth. (**B**,**C**) Box and whisker plots of % change of area of individual organoids treated with DMSO (**B**) or ARQ-092 (**C**). (**D**) Representative photos of organoids containing *Cdh1*-null cells exposed to DMSO, ARQ-092, or MK2206 on day 2 and day 6 of growth. (**E**) Box and whisker plots of % change of area of individual organoids treated with MK2206. (**F**) Bar graph showing average % change in area of organoids exposed to DMSO, ARQ-092, or MK2206 for 96 h. (**G**) Bar graph showing the change in area of drug-treated organoids normalised to DMSO-treated organoids. (For all graphs, error bars = SEM; ns: *p* > 0.05, *: *p* < 0.05, **: *p* < 0.01, ***: *p* < 0.001; *n* = 3 independent biological replicates; unpaired two-sided *t*-test.)

The DMSO-treated wild-type (WT) and *Cdh1*-deleted organoids displayed normal growth and no signs of death (Figure 4A,D). They increased in size significantly between day 2 and day 6 and, by day 6, showed no signs of disintegration, flattening, or substantial darkening. After 96 h treatment with 5 µM ARQ-092, the WT organoids displayed relatively normal growth, although some showed darkening on both days 2 and 6 (Figure 4A). In contrast, *Cdh1*-deleted organoids that were exposed to ARQ-092 exhibited distinct death phenotypes (Figure 4D). By day 6, these organoids had lost their transparency, and were instead comprised of darkened, grainy tissue. This darkening indicated dying cells, and the grainy texture along with the disrupted borders suggested that the organoids were beginning to lose their structure and break down.

We also tested MK2206 efficacy in our organoid model. WT organoids that had been exposed to MK2206 (6.25 µM), showed no obvious signs of death by day 6, 96 h after initial drug exposure (Figure 4A). However, the MK2206-treated *Cdh1*-deleted organoids showed a marked decrease in growth rate and general health of the organoids (Figure 4D).

Change in organoid area was used as the primary quantitative measure of drug effectiveness. To measure this, the total area of each organoid was calculated on day 2 and day 6, and the difference between the two calculated as a percentage change. By using percentage change, we were able to account for the relatively high levels of variation in growth rate and starting size of the organoids. These values (from all three replicates) were then plotted on a line graph, where each line represents one organoid (Figure 4B,C,E).

Both WT and *Cdh1*-deleted organoids treated with DMSO displayed normal growth and increased at comparable rates (Figure 4B). However, both the WT and *Cdh1*-deleted organoids grew at a significantly reduced rate after exposure to ARQ-092 (Figure 4C). Notably, there were a large number of *Cdh1*^−/−^-deleted organoids displaying less than a 50% total increase in size after ARQ-092 treatment. The average percent change in area across all organoids was calculated and plotted on a bar graph (Figure 4F). Over 96 h of treatment, WT organoids exposed to ARQ-092 had on average an 126% increase in size, whereas *Cdh1*-deleted organoids showed a 45% average increase in size (*p* = 0.012).

The WT organoids exposed to MK2206 grew at a slightly reduced rate compared to DMSO-treated controls; however, the *Cdh1*-deleted organoids exposed to MK2206 showed a striking reduction in growth rate (Figure 4F). Over 96 h of treatment, WT organoids exposed to MK2206 had on average an 185% increase in size, whereas *Cdh1* knock-down organoids showed only a 52% increase in size (*p* = 0.0005).

In order to account for the (non-significant) difference between the growth rates of WT and *Cdh1*-deleted organoids treated with DMSO, we normalised organoid change in area after drugs relative to DMSO (Figure 4G). After ARQ-092 treatment, WT organoids were on average 0.43-fold the size of DMSO controls, whereas *Cdh1*-deleted organoids were 0.19-fold the size. However, due to relatively high variation, this result was not significant (*p* = 0.1). After MK2206 treatment, WT organoids were 0.6-fold the size of DMSO controls, whereas *Cdh1*-deleted organoids were 0.23-fold the size (*p* = 0.005). Therefore, allosteric inhibition of AKT demonstrates a significant synthetic lethal effect in our *Cdh1*-deleted organoids.

### 2.5. E-cadherin Expression Affects AKT Isoforms Differently in Breast and Gastric Cells (or in Normal and Cancer Cells)

**Figure 5:**
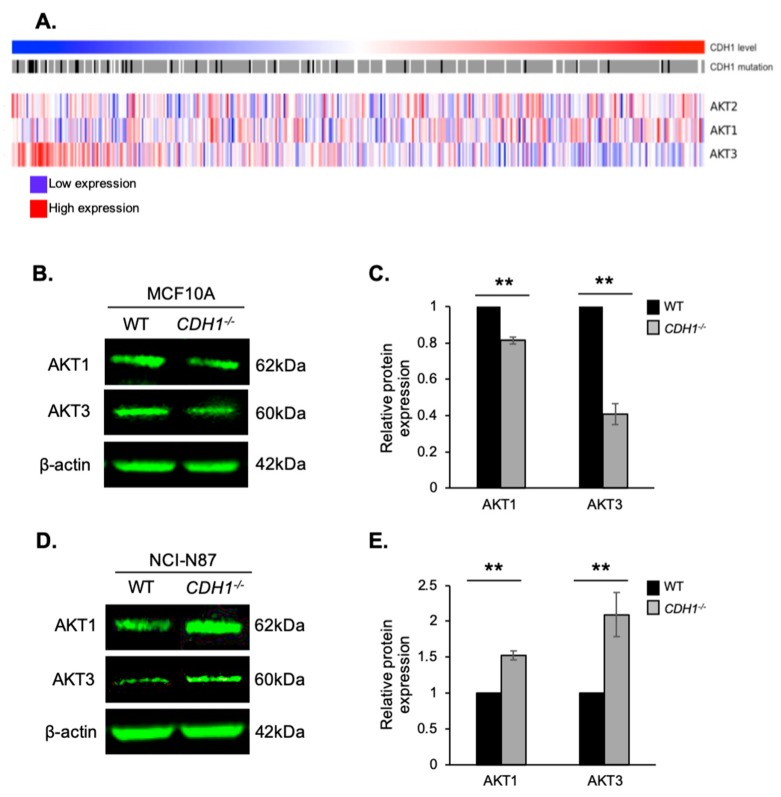
AKT isoforms are differentially regulated in *CDH1*-null gastric tumours, as well as non-malignant breast and gastric cancer cells. (**A**) Gene expression analysis of *AKT* isoforms and *CDH1* in gastric cancer samples from the TCGA. Samples ordered by *CDH1* expression level: Low to high (left to right). *CDH1* mutations: Light grey, wild-type; dark grey, silent somatic mutation; black, somatic mutation. (**B**) Protein expression of AKT1 and AKT3 in MCF10A-WT and MCF10A-*CDH1*^−/−^ cells. (**C**) Relative expression of AKT1 and AKT3 protein in MCF10A-WT and MCF10A-*CDH1*^−/−^ cells. (**D**) Protein expression of AKT1 and AKT3 in NCI-N87-WT and NCI-N87-*CDH1*^−/−^ cells. (**E**). Relative expression of AKT1 and AKT3 protein in NCI-N87-WT and NCI-N87-*CDH1*^−/−^ cells. (For all graphs, error bars = SEM; ** = *p* < 0.01; *n* ≥ 3 independent biological replicates; unpaired two-sided *t*-test.)

To explore the relationship between *AKT* isoform and *CDH1* expression in gastric cancer, we performed an analysis of gene expression data from a stomach cancer database containing information from 415 tumours (TCGA, STAD cohort) (Figure 5A). In these gastric tumour samples, there was a non-significant correlation between *AKT1* and *CDH1* expression (correlation 0.081, *p* = 0.1). Conversely, *AKT3* expression was significantly negatively correlated with *CDH1* expression (correlation −0.439, *p*-value < 2.2 × 10^−16^). There was no correlation between *AKT2* and *CDH1* expression in these tumour samples.

To further investigate the relationship between the loss of *CDH1* on AKT isoforms 1 and 3 in breast and gastric cancer cells, we measured the isoform protein expression in our cells. Both AKT1 and AKT3 protein levels were significantly decreased in MCF10A-*CDH1*^−/−^ cells compared with WT cells (0.81- and 0.41-fold expression, respectively) (Figure 5B,C). In contrast, expression of the AKT1 and AKT3 isoforms were significantly increased in NCI-N87-*CDH1*^−/−^ cells compared with NCI-N87-WT cells (1.52- and 2.09-fold, respectively) (Figure 5D,E). In general, increases in AKT expression are associated with enhanced cell survival; however, in certain circumstances, such as was seen in a model of ischaemia/reperfusion injury [29], AKT may induce apoptosis via feedback inhibition of PI3K.

## 3. Discussion

In this study, we have demonstrated that breast and gastric cells lacking E-cadherin exhibit an enhanced sensitivity to cell death mediated by allosteric AKT inhibitors.

Importantly, there was a significant preferential priming of MCF10A-*CDH1*^−/−^ cells for apoptosis after drug treatment, and this priming correlated well with the actual induction of apoptosis. Of the four allosteric AKT inhibitors tested, MK2206 and ARQ-092 exhibited the most consistent synthetic lethal relationship with E-cadherin in both breast and gastric cells. These drugs were selected to be tested in our novel gastric organoid model system—where both drugs preferentially inhibited the growth of organoids with *Cdh1* conditional deletion compared with wild-type controls.

pAKT, pan-AKT, and isoforms 1 and 3 are downregulated in MCF10A cells lacking *CDH1*, but are upregulated in *CDH1*-deficient NCI-N87 cells. We hypothesise that in MCF10A-*CDH1*^−/−^ cells, their sensitivity to allosteric inhibitors is due to the very low levels of active AKT. It is well documented that AKT phosphorylation of the proapoptotic BAD protein [30] releases BAD binding of Bcl-xL and prevents subsequent cytochrome C release from mitochondria, thereby inhibiting intrinsic apoptosis [31]. Decreased AKT expression in MCF10A-*CDH1*^−/−^ may be sensitising the cells to drug-induced apoptosis through this mechanism.

Conversely, we postulate that despite having significantly higher levels of AKT protein (pan, phospho, and AKT1 and 3), the NCI-N87-*CDH1*^−/−^ cells maintain sensitivity to allosteric inhibition of AKT because of an enhanced dependence on AKT signalling. Potentially, the upregulation of the pro-survival and pro-proliferative AKT signalling pathway may be a response to death signals being activated in these cells subsequent to the loss of *CDH1* expression and the acquisition of malignancy [32]. Loss of E-cadherin in cancer cells has been shown to activate AKT signalling as one mechanism to acquire resistance to anoikis subsequent to EMT [33,34].

Interestingly, the dual AKT-PDK1 allosteric inhibitor, PHT-427, exhibited no synthetic lethal effect in our MCF10A-*CDH1*^−/−^ cells. While PDK1 is critical for the activation of AKT, via phosphorylation of Threonine 308 [35], it also acts as a “master kinase” with many documented targets [36]. The inhibition of PDK1 may have AKT-independent effects which interfere with the synthetic lethal effect of AKT inhibition alone.

AKT is postulated to contribute to gastric carcinogenesis and to influence prognosis [37], and our bioinformatic analysis of the TCGA dataset showed that there was significant upregulation of *AKT3* RNA in *CDH1*-deficient tumours, but variable *AKT1* expression. Interestingly, in a recent proteomic study, upregulation of AKT pathways was found in *CDH1*-mutated diffuse gastric cancer tumours [13]. Therefore, AKT may represent an attractive drug target for gastric cancers with inactivating *CDH1* mutations.

Of the AKT inhibitors that have been developed for cancer treatment, the allosteric inhibitors used in this study are some of the most clinically advanced [38]. MK2206 has been shown in numerous studies to be well tolerated, but has exhibited limited efficacy as a monotherapy in a variety of solid tumours, such as gastric [39] and colorectal [40] cancers. However, trials of MK2206 in combination with traditional chemotherapeutic agents, such as paclitaxel, have had more success [41]. Additionally, MK2206 appears to synergise with EGFR/HER2 inhibition, and preliminary anti-tumour activity of MK2206 in combination with trastuzumab [42] has been documented. This leads to the possibility that MK2206 could be used in combination with other drugs to target diffuse gastric cancers with *CDH1* mutations.

ARQ-092 has been shown to exhibit anti-tumour activity in a variety of solid and haematological tumours harbouring activating PIK3CA mutations [43], and promising clinical activity in patients harbouring AKT^E17K^ mutations [44]. Encouraging anti-cancer activity was also demonstrated after ARQ-092 treatment in ovarian cancer patients previously treated with carboplatin and paclitaxel [45]. ARQ-092 is additionally being investigated for the primary treatment of Proteus syndrome, an overgrowth disorder resulting from an activating mutation in *AKT1* (AKT^G49A^) [46]. Interestingly, the goals of the treatment of disorders such as Proteus syndrome would potentially align with our criteria for a chemopreventative strategy for patients lacking *CDH1*. Minimal toxicity is essential if ARQ-092 is to be used long-term. The first study to clinically evaluate tolerated ARQ-092 dose in adults and children with Proteus syndrome has just been published [47]. In general, the drug was well tolerated, with mostly grade 1 and 2 adverse effects. Importantly, ARQ-092 reduced phosphorylated AKT in affected tissues of most of the patients, as well as induced an apparent arrest of the cerebriform connective tissue nevus (CCTN) lesions present in the patients.

In conclusion, our study has demonstrated that cell lines (MCF10A and NCI-N87) and mouse-derived gastric organoids lacking *CDH1* are more sensitive to the apoptotic effects of allosteric AKT inhibitors. Bioinformatics analysis indicates that there is a strong correlation between the loss of *CDH1* and an increased expression of *AKT3* in gastric cancer. This has revealed a vulnerability in diffuse gastric cancer to AKT inhibition and we propose that this will lead to new chemoprevention and chemotherapy strategies for hereditary and sporadic diffuse gastric and lobular breast cancer patients.

## 4. Materials and Methods

### 4.1. Cell Culture

MCF10A cells (CRL 10317), a non-tumorigenic mammary epithelial cell line, and the derived isogenic line with *CDH1* knockout (MCF10A-*CDH1*^−/−^) were purchased from Sigma (#CLLS1042). The MCF10A isogenic lines were cultured in DMEM/F12: (1:1) (Invitrogen, Carlsbad, CA, USA) with 5% horse serum (Invitrogen), 10 μg/mL Actrapid neutral insulin (Novo Nordisk Pharmaceuticals Ltd. Bagsværd, Denmark), 20 ng/mL human epidermal growth factor (Peprotech, Rehovot, Israel), 100 ng/mL cholera toxin, and 500 ng/mL hydrocortisone (Sigma Aldrich, St Louis, MO, USA). NCI-N87 gastric cancer cells (CRL-5822) were purchased from ATCC and *CDH1* knockouts were generated via Crispr-Cas9 (manuscript in preparation). Briefly, the NCI-N87-*CDH1*^−/−^ cell line was generated using the following CRISPR guide RNA sequence: 5′ GCTTCATTCACATCCAGCACATCCACGGTGAC 3′ which targeted exon 10 of the *CDH1* gene. This gave rise to a single nucleotide frameshift deletion followed by a T/A SNP in the *CDH1* gene, which was confirmed by Sanger sequencing. The NCI-N87 isogenic lines were cultured in DMEM/F12 (1:1) (Invitrogen) with 10% fetal bovine serum (Invitrogen). All cells were grown at 37 °C with 5% CO_2_.

### 4.2. Small Molecule Inhibitors and Chemicals

The AKT inhibitors ipatasertib (#S2808), AZD5363 (#S8091), MK2206 (#S1078), SC66 (#S5313), and the BH3 mimetic ABT-737 (#S1002) were purchased from SelleckChem (Houston, TX, USA). ARQ-092 (#21388), perifosine (#1008112), PHT-427 (#24188), staurosporine (#81590), digitonin (#14952), and FCCP (#15218) were purchased from Cayman Chemical. Oligomycin (#O4876), β-mercaptoethanol (#M3148), and endoxifen (E8285) were purchased from Sigma Aldrich (St Louis, MO, USA).

### 4.3. Nuclei Enumeration Assay

MCF10A-WT/MCF10A-*CDH1*^−/−^ and NCI-N87/NCI-N87-*CDH1*^−/−^ cells were seeded in 96-well black plates (Greiner, Kremsmünster, Austria) at 4 (MCF10A) or 10 (NCI-N87) × 10^4^ cells per well and left to attach overnight. The next day, outer wells of plates were stained with 1 µg/mL Hoechst 33,342 in PBS for 30 min and imaged on a Cytation 5 imager (Biotek, Winooski, VT, USA). Nuclei were counted at this stage to ensure plating accuracy. If the ratio of WT:*CDH1*^−/−^ cells was >0.65 and <1.3, then plates proceeded to drugging. Cells were drugged with a dose response of compounds as previously described [48] and incubated for a further 48 h, cells were then fixed and stained with 1 µg/mL Hoechst 33,342, 0.25% paraformaldehyde and 0.075% saponin in PBS. Six fields/well at 4× magnification were captured using the Cytation 5 imager (Biotek). Nuclei were counted using Gen5 software (Biotek) and normalised to the vehicle control for each cell line.

### 4.4. Western Blotting

MCF10A-WT and *CDH1*^−/−^ cells (4 and 5 × 10^5^ cells/plate) or NCI-N87-WT and *CDH1*^−/−^ (1 and 1.3 × 10^6^ cells per plate) cells were seeded in 10 cm plates and left to attach overnight. Next, cells were left untreated or exposed to DMSO or drug treatment for 4 h in the incubator. Following drug treatment, plates were washed twice with ice cold TBS and stored at −80 °C for ≥1 h. Cells were then scraped into 120 µL of lysis buffer (25 mM HEPES, 100 mM NaCl, 1 mM EDTA, 10% (*v*/*v*) glycerol, 1% (*v*/*v*) Triton-X-100) containing Complete Mini Protease Inhibitor tablets (Roche, Basel, Switzerland). Tubes containing cell lysates were held on ice for 30 min, vortexing every 5 min, and then spun at 14,000 rpm for 5 min at 4 °C. The supernatant was moved to a clean tube and the protein concentration measured by BCA Assay (Thermo Fisher Scientific, Waltham, MA, USA). Next, 20 µg of protein was mixed with 2X Laemmli buffer and run on 12% SDS-Page gels. After transferring to nitrocellulose membranes, they were blocked with 5%-BSA or milk in TBS-tween for 1 h and incubated overnight with primary antibodies at 4 °C. Antibody concentrations: p-AKT Ser473 (Cell Signalling #4060) at 1:1000, pan-AKT at 1:1000 (Cell Signalling #2920), AKT1 at 1:1000 (Cell signalling #75692), AKT3 at 1:1000 (Cell Signalling #14982), E-cadherin 1:1000 (RnD Systems #AF748), and B-actin 1:2500 (Sigma Aldrich #A2066). Fluorescent secondary antibodies anti-rabbit (LI-COR #925-32211), anti-goat (LI-COR #925-32214), and anti-mouse (LI-COR #925-32210) were used at 1:15,000. Membranes were imaged on LI-COR Odyssey Imaging System (Lincoln, NE, USA).

### 4.5. FACS

MCF10A and MCF10A-*CDH1*^−/−^ cells were seeded at 4 and 5 × 10^4^ cells per well in 6-well plates. The next day, cells were drugged with AKT inhibitors and left for 72 h. The day before analysis, control wells were drugged with Staurosporine (0.1 µM). On the day of analysis, cells were trypsinised, spun down at 1500 rpm for 5 min, and resuspended in 500 µL Annexin-binding buffer (10 mM Hepes (pH 7.4), 140 mM NaCl, 2.5 mM CaCl_2_). Cells were stained with propidium iodide (Sigma Aldrich, St. Louis, MO, USA) and FITC-Annexin-V (#556420, BD Biosciences, San Jose, CA, USA) for 15 min in the dark. Samples were analysed on BD Fortessa Flow cytometer. Compensation controls were: Untreated unstained, heat-treated cells stained with PI, and Staurosporine (0.1 µM)-treated cells stained with FITC-Annexin-V.

### 4.6. Apoptosis Priming

Our protocol for measuring apoptosis priming was based upon that described in Montero et al. [49]. MCF10A-WT and MCF10A-*CDH1*^−/−^ cells were seeded at 1.5 and 1.8 × 10^5^ cells per well in 6-well plates and left to attach overnight. The cells were then drugged with AKT inhibitors AZD5363 (10 µM), ARQ-092 (2 µM), MK2206 (6.25 µM), perifosine (7.5 µM), SC66 (0.78 µM), the PI103 inhibitor PI103 (1 µM), or the BH3 mimetic drug ABT-737 (10 µM). DMSO (0.1%) was used as a vehicle control. Following 16–20 h treatment, cells were trypsinised, counted, and washed with M-EB buffer (150 mM Mannitol, 10 mM HEPES-KOH pH 7.7, 50 mM KCl, 0.02 mM EGTA, 0.02 mM EDTA, 0.1% BSA, 5 mM succinate). Cells were then stained with a 4× concentrated staining solution containing 4 mM JC-1, 40 mg/mL oligomycin, 0.02% digitonin, 20 mM 2-mercaptoethanol in M-EB. This cell/dye solution was left to stand at room temperature for 10 min in the dark to allow permeabilisation and dye equilibration. After staining, 15 µL (containing 15,000 cells) was deposited per well in a black 384-well plate (Greiner, Kremsmünster, Austria) already containing 2× concentrated solutions of BIM BH3 peptide (0.6 µM, purchased from New England Peptide) or FCCP (12.5 µM) in ME-B. The plate was briefly shaken and equilibrated to 30 °C inside the CLARIOstar microplate reader (BMG Labtech, Ortenberg, Germany) and fluorescence at 590 nm monitored every 5 min for 65 min. Percentage loss of mitochondrial membrane polarisation after drug treatment and exposure to BIM peptide was calculated by normalisation to the solvent only control DMSO (0% depolarisation) and the positive control FCCP (100% depolarisation). Firstly, area under the curve (AUC) for each condition was calculated, followed by calculation of the depolarisation using the equation:

Depolarisation = 1 (AUC_sample_ − AUC_FCCP_)/(AUC_DMSO_ − AUC_FCCP_)

Individual depolarisation analysis was performed using 3–5 technical replicates for each control and sample.

### 4.7. Organoid Culture

Organoids were generated using the stomachs of neonatal mice and an inducible Cre-lox system [50], controlling both *Cdh1* deletion and the activation of the fluorescent marker protein TdTomato under the CD44 stem cell promotor (*CD44*-Cre/*Cdh1*(^fl/fl^)/TdTomato). Single loxP sites flank a region encompassing exons 6 through 10 of Cdh1, followed by a floxed selection cassette which was excised via in vitro Cre mediated recombination. Mice were used 1–2 days post-birth. Stomach tissue was removed and washed thoroughly in PBS with gentamycin before being minced to <0.5 mm^3^ segments. Organoids were cultured in an air–liquid interface (ALI) system with myofibroblast co-culture (protocol adapted from [51]). Briefly, two separate 1.2 mL layers of collagen (Novachem, Calgary, AB, Canada) matrix were prepared inside a transwell insert (Corning, Corning, NY, USA), with both layers containing 5 × 10^5^ myofibroblasts, and the top layer containing stomach tissue fragments. This transwell insert was placed inside a 35 mm dish, with 3 mL of growth medium added to the external dish. Organoid growth medium comprised F-12, 20% FBS, and 50 µg/mL Gentamycin. Organoids were grown at 37 °C with 5% CO_2_. Knockout of *Cdh1* was induced by addition of endoxifen (5 µM in DMSO) to the medium on day 0 post-seeding. An equivalent volume of DMSO was used for control wells. Growth of organoids was monitored daily by brightfield microscopy (Eclipse Ti Inverted Microscope System, Nikon, Tokyo, Japan).

### 4.8. Drug Treatment of Organoids

For each drug screen, organoids were established as described above. After 48 h in organoid growth medium containing endoxifen or DMSO, medium was replaced with media containing drug and/or DMSO. Brightfield images of the organoids were captured every 24 h (after plating) until 96 h (day 6) to monitor organoids. Images of individual organoids were assessed for changes in morphology and size, measured as area. Percentage area change per organoid from day 2 to day 6 was calculated. Significance was assessed by Wilcoxon rank sum test.

### 4.9. RNA-seq Data

The correlation analysis between *AKT* (isoforms 1, 2, and 3) and *CDH1* expression levels was assessed using RNA-seq data from the Stomach Adenocarcinoma (STAD) cohort in The Cancer Genome Atlas. Gene-level count data were downloaded from the International Cancer Genome Consortium data portal (https://dcc.icgc.org/releases/release_25) for 415 tumour samples, and normalised using the voom [52] methodology in the limma package for R [53]. The heatmap of AKT expression levels was generated using the gplots [54] package for R (version 3.4).

## 5. Conclusions

We have identified that breast cells, gastric cancer cells, and mouse-derived organoids that are E-cadherin-deficient have increased sensitivity to the allosteric AKT inhibitors ARQ-092 and MK2206. The identification of this synthetic lethal relationship may lead to new treatment strategies for hereditary and sporadic cancers with mutations in the *CDH1* gene.

## Supplementary Materials

The following are available online at https://www.mdpi.com/2072-6694/11/9/1359/s1: Supplementary method: Immunofluorescence of organoids; Figure S1: E-cadherin is not expressed in MCF10A-*CDH1*^−/−^ or NCI-N87-*CDH1*^−/−^ cells; Figure S2: Allosteric AKT inhibitors preferentially induce apoptosis in *CDH1*-null breast cells; Figure S3: E-cadherin expression in wild-type organoids; Figure S4: Assessment of percentage of Cdh1-null cells in a sample of organoids.
