# Supplementary material for: Allosteric AKT Inhibitors Target Synthetic Lethal Vulnerabilities in E-Cadherin-Deficient Cells
Source: Cancers (Basel). 2019 Sep 13;11(9):1359. doi: 10.3390/cancers11091359 (PMC6769709; doi:10.3390/cancers11091359)

Supplementary Information

Method: Immunofluorescence of organoids

Segments containing organoids of interest were removed with minimal collagen surrounding. In a 1.5ml tube, 1-2 segments were fixed with 4% paraformaldehyde in PBS before multiple PBS washes. Blocking and permeabilisation was performed in PBS containing 10% FHS and 0.5% TritonX for 60 minutes at room temperature with agitation. Organoids were then incubated in primary antibody (E-Cad: RnD: AF748 at 1:100) for 60 minutes in PBS with 10% FHS and 2% FBS before incubation with secondary antibody (Donkey anti-goat (ThermoFisher #A11055) at 1:1000 dilution in the same buffer), overnight at $4^{o}C$. Multiple washes were performed in PBS before ProLong Gold antifade reagent with DAPI (Thermo Fisher) added to the tubes. Organoid morphology was preserved by utilising a bridge mounting method. Organoids were imaged on an Olympus Fluroview Confocal Microscope. Approximate knockout efficacy percentage was determined by calculating the total area of E-cadherin deficient cells (no green E-cadherin staining) as a percentage of the total area of the organoid.

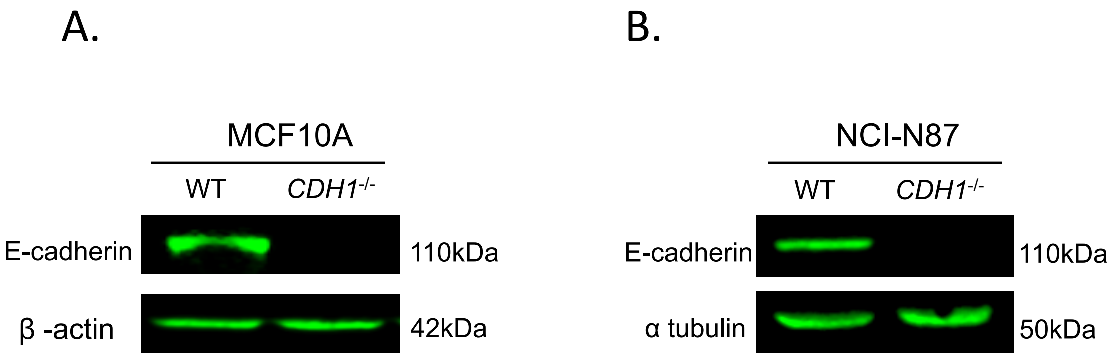

Supplementary figure 1: E-cadherin is not expressed in MCF10A-*CDH1*$^{-/-}$ or NCI-N87-*CDH1*$^{-/-}$ cells. (**A/B**) Western blot analysis of E-cadherin expression in isogenic cell lines generated from MCF10A (**A**) and NCI-N87 (**B**) cells.

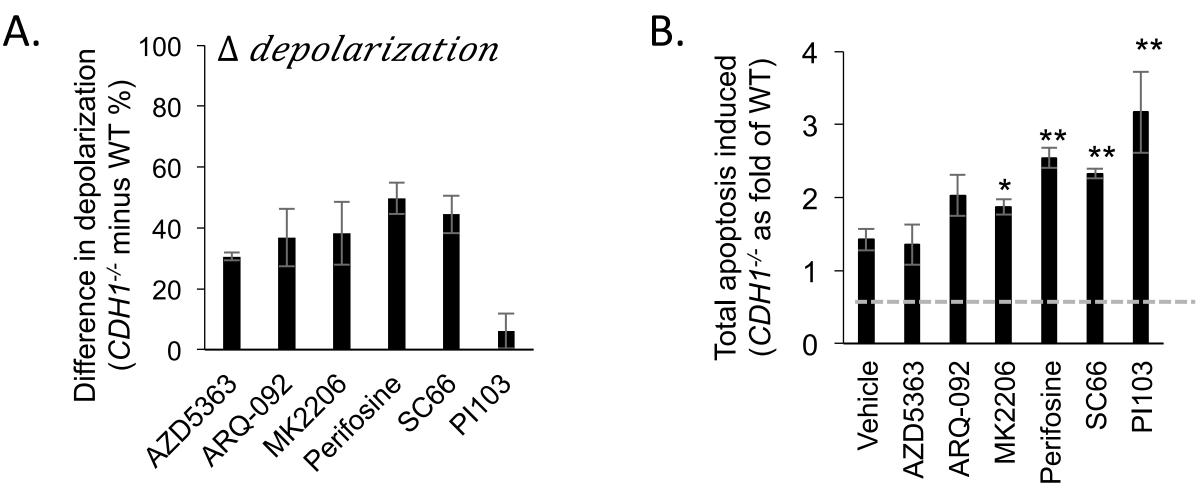

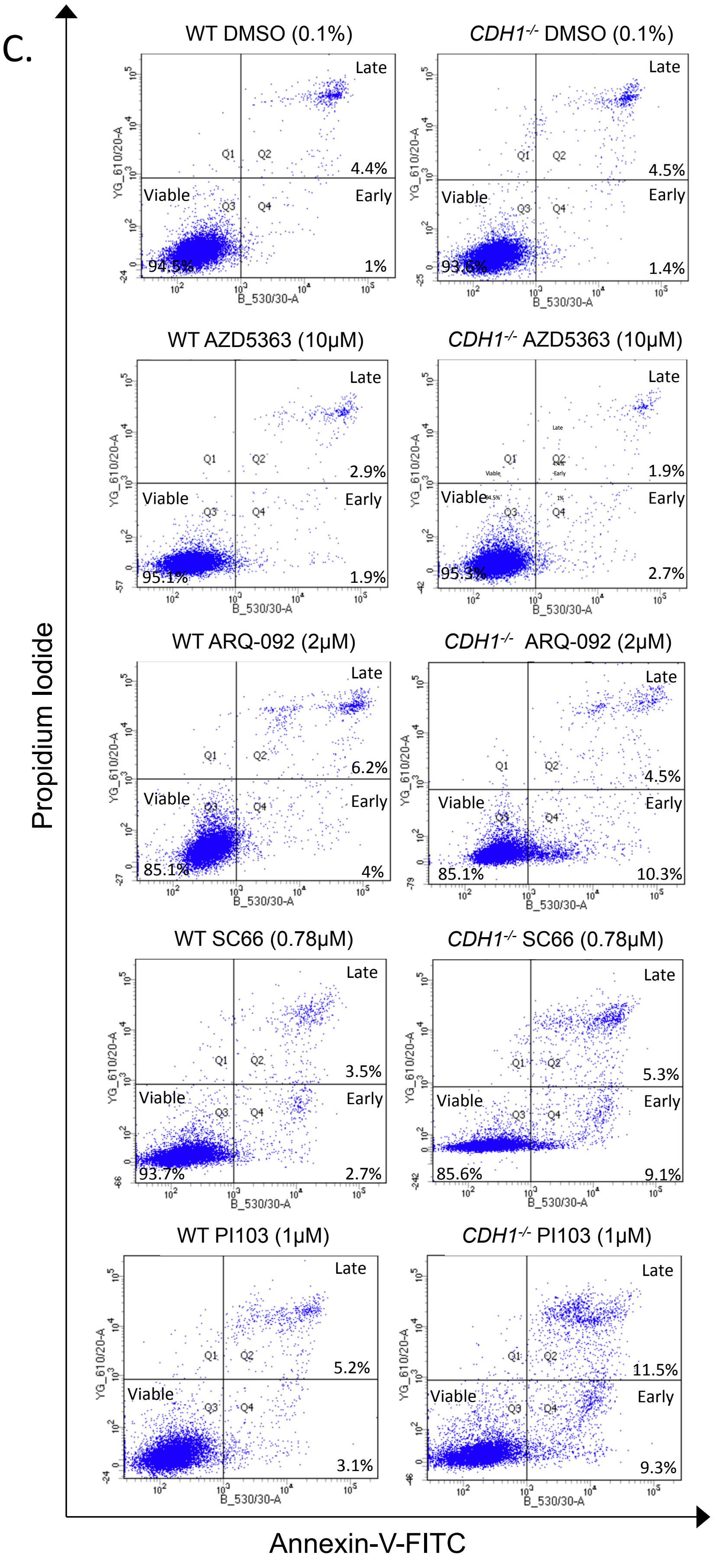

Supplementary figure 2. Inhibition of AKT preferentially primes *CDH1*-null cells for apoptosis. (**A**) Difference in mitochondrial membrane depolarization between WT and *CDH1*$^{-/-}$ cells after drug treatment (*CDH1*$^{-/-}$ depolarisation minus WT depolarization). (**B**) Total apoptosis of *CDH1*$^{-/-}$ cells normalised to WT apoptosis levels after the same treatments. (**C**) Representative histograms of drug treated MCF10A-WT and *CDH1*$^{-/-}$ cells stained with Annexin-V-FITC and propidium iodide and analysed on BDFortessa. (For all graphs, error bars = SEM; ns = P>0.05, * = P<0.05, ** = P<0.01, *** = P<0.001; n ≥ 3 independent biological replicates; unpaired two-sided t-test).

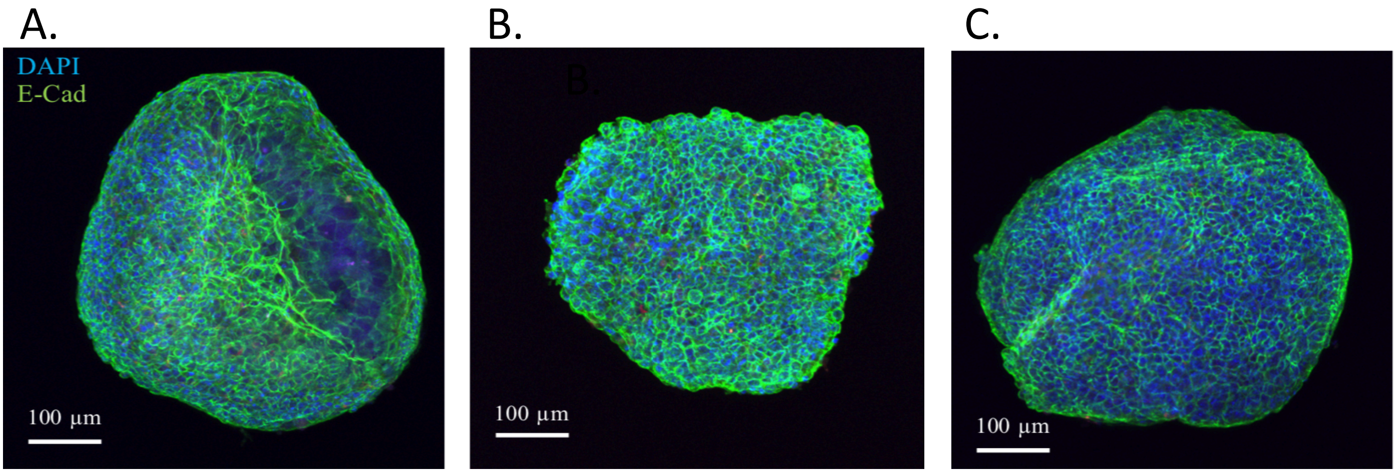

Supplementary figure 3: E-cadherin expression in wildtype organoids. A/B/C. Confocal microscopy images of three typical organoids fixed at day 5 that have not been exposed to endoxifen in the media. Wildtype organoids show consistent E-cadherin expression.

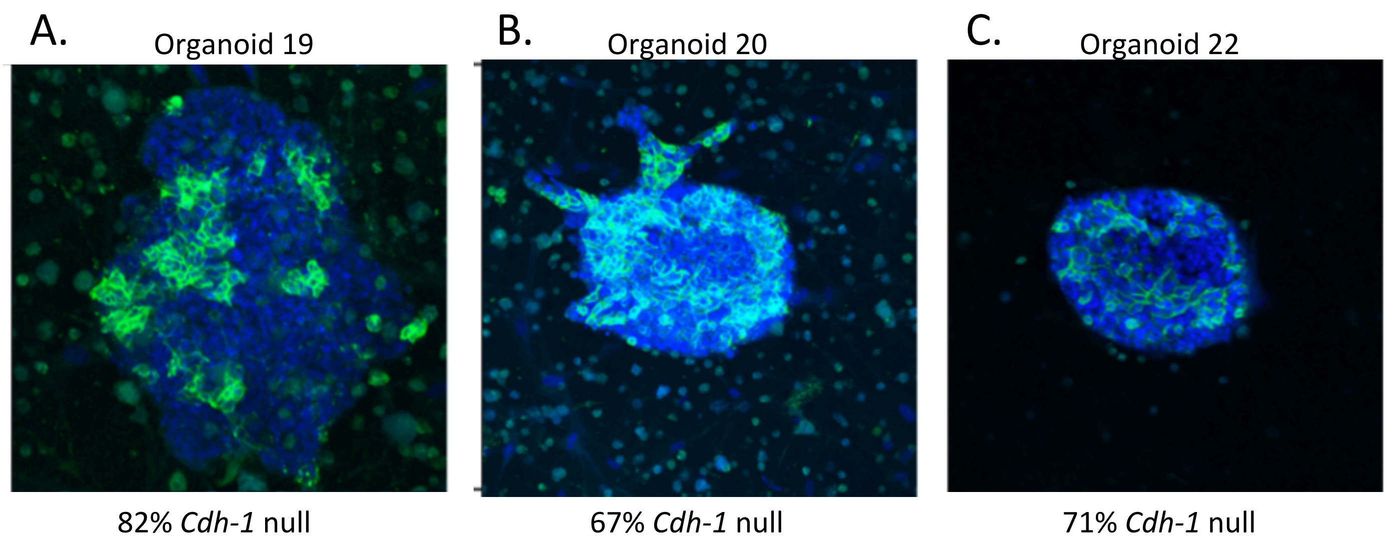

Supplementary figure 4: Assessment of percentage of *Cdh1*-null cells in a sample of organoids. Organoids were generated, induced with endoxifen for 5 days. A/B/C. After immunofluorescence and confocal imaging, the approximate knockout efficiency percentage was determined by calculating the total area of E-cadherin deficient cells (no green E-cadherin staining) as a percentage of the total area of the organoid. Organoid 20 (B) displays slightly different colouring due to the overlay of green and blue staining in different cell layers within the organoid.

WESTERN BLOTS

FIGURE 1. MCF10A isogenic cell lines

G.

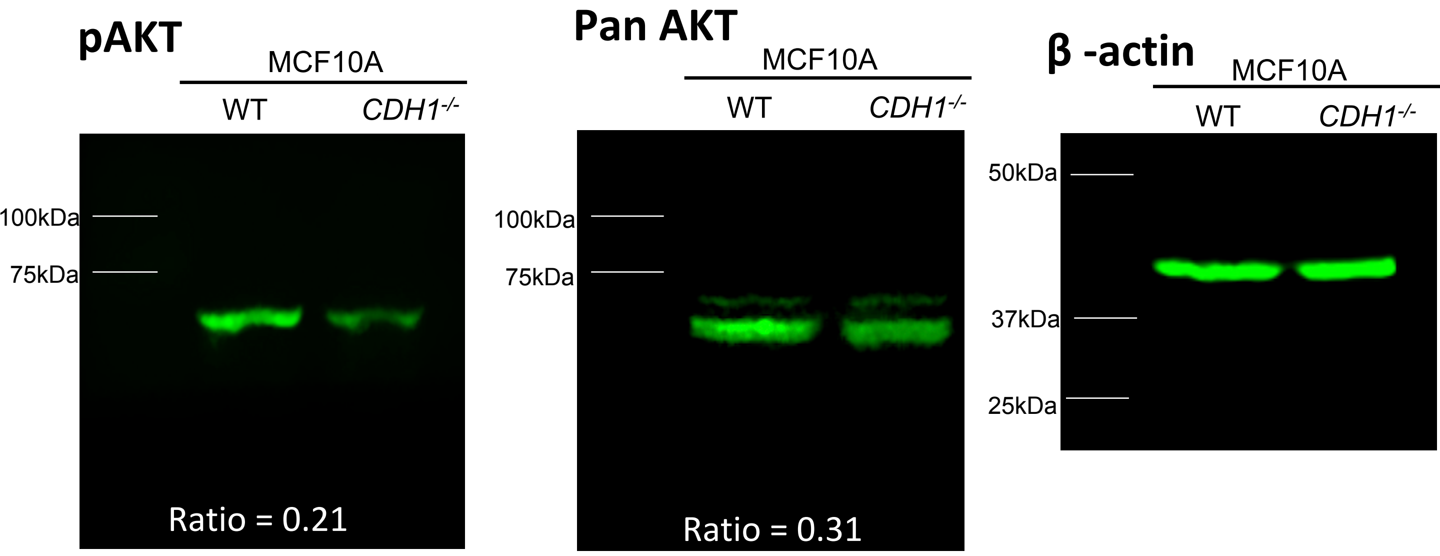

**Ratios are normalized to β-actin*

I.

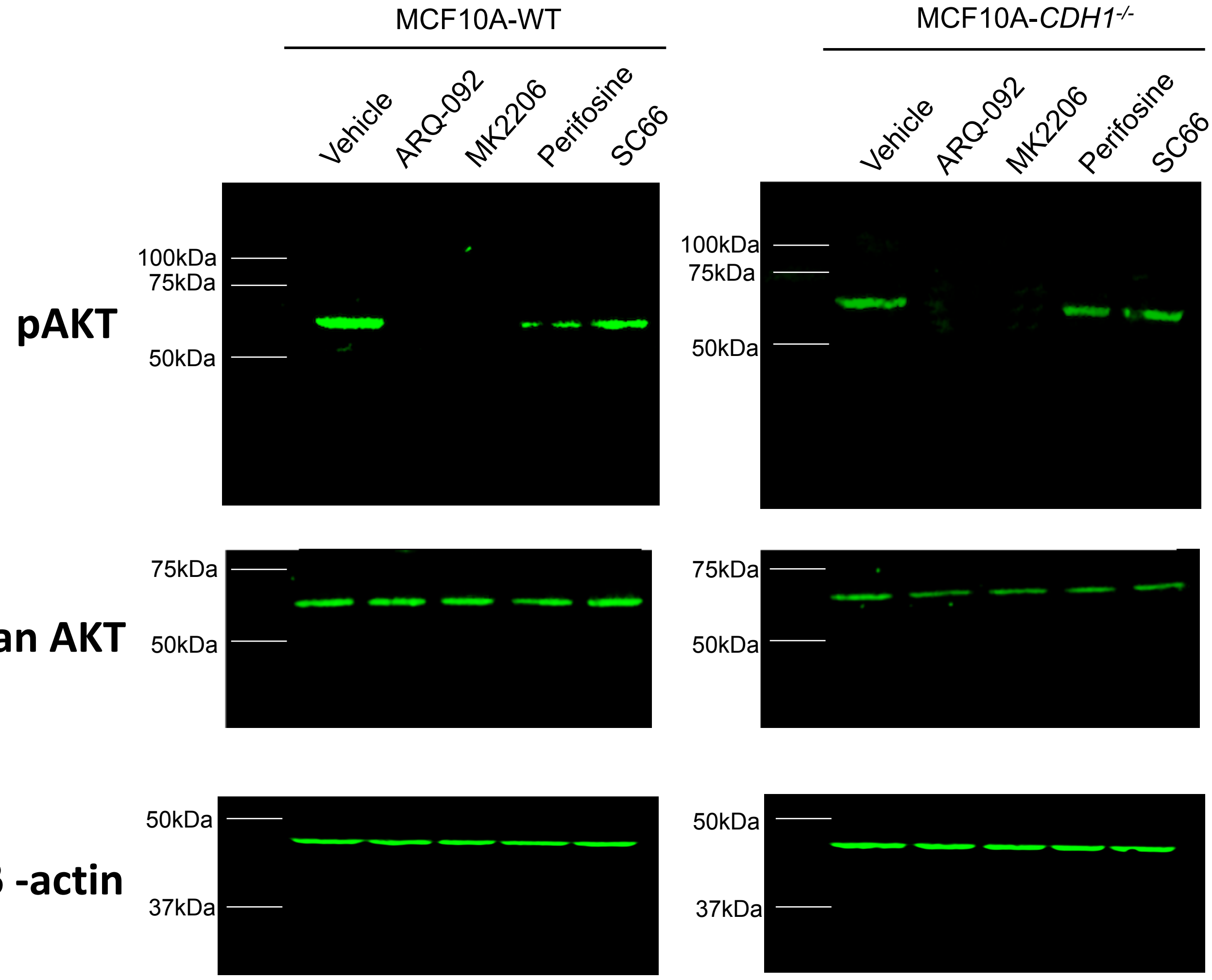

FIGURE 3. N87 isogenic

A.

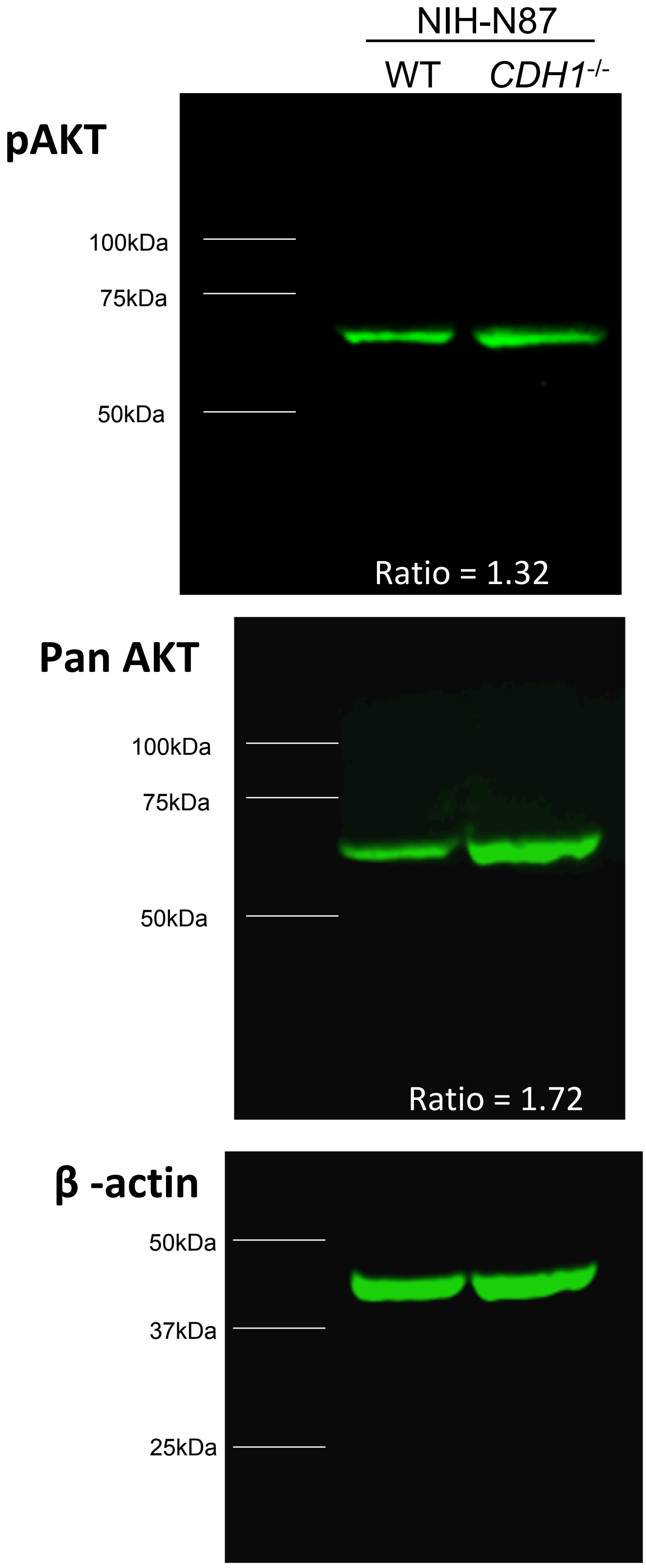

**Ratios are normalized to β-actin*

FIGURE 5. AKT isoforms

B.

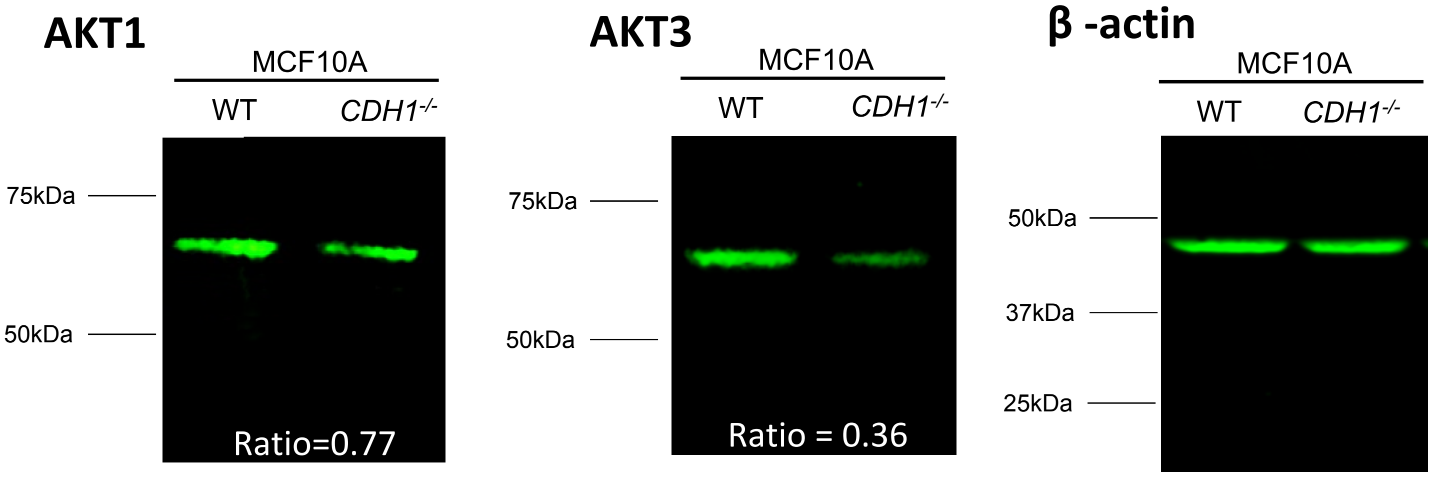

D.

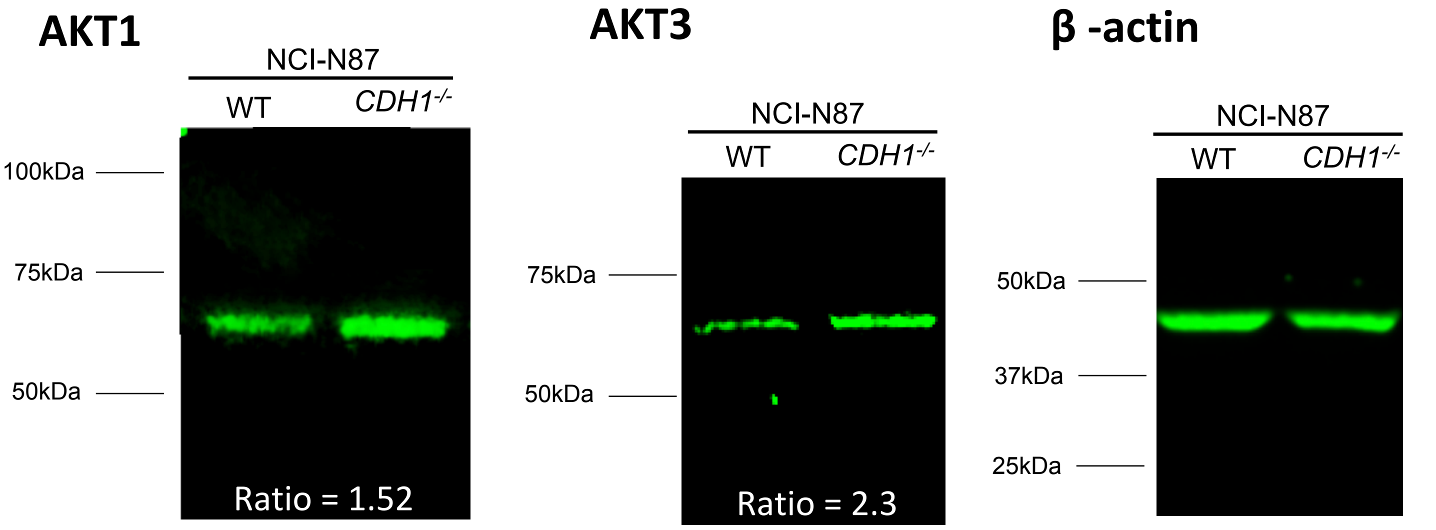

**Ratios are normalized to β-actin*

Supplementary 1. E-cadherin expression in MCF10A and NCI-N87 isogenic cell lines treated

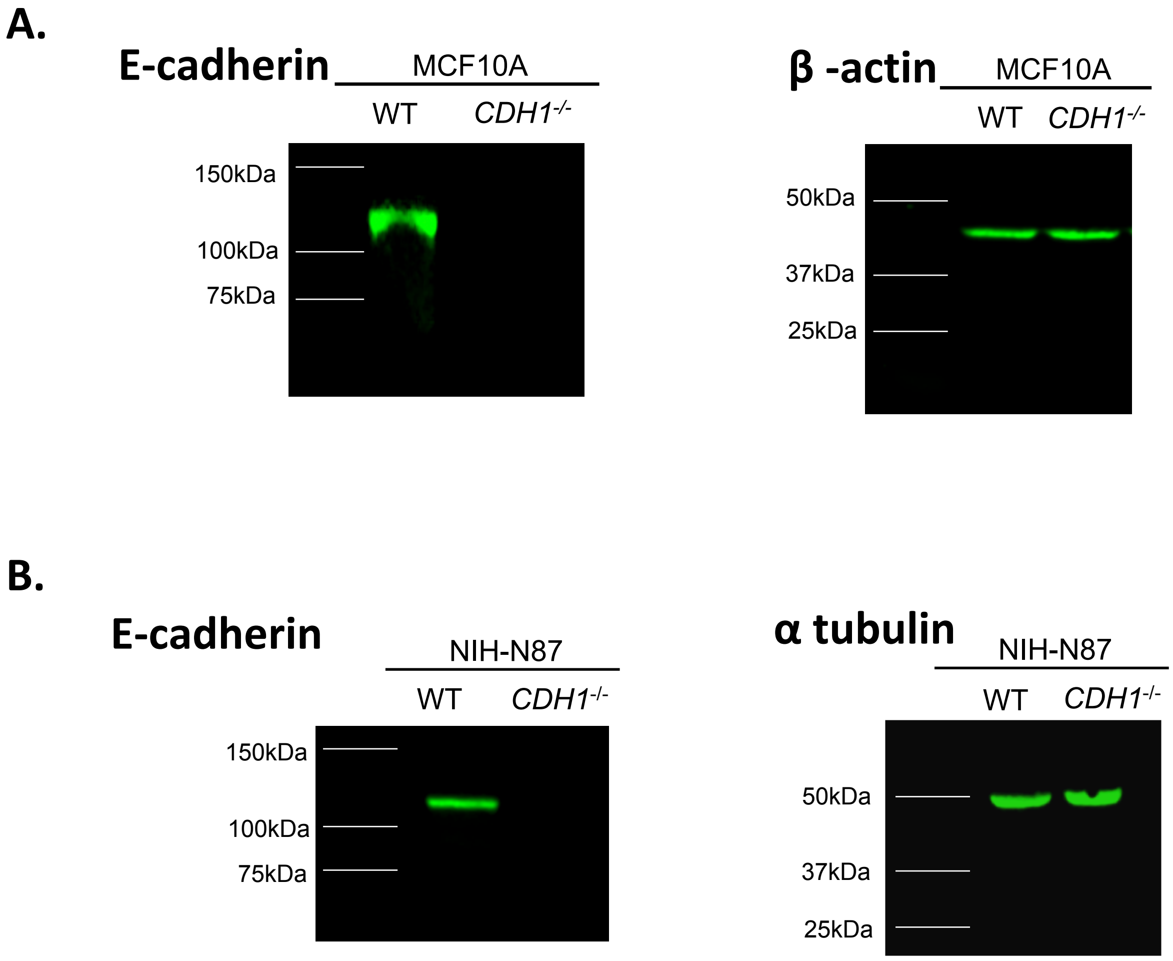

Supplement: Supplementary file 1 [file cancers-11-01359-s001.pdf]
